# Improving public health control of schistosomiasis with a modified WHO strategy: a model-based comparison study

**DOI:** 10.1016/S2214-109X(19)30346-8

**Published:** 2019-10-01

**Authors:** Emily Y Li, David Gurarie, Nathan C Lo, Xuewei Zhu, Charles H King

**Affiliations:** 1 School of Medicine, Center for Global Health and Diseases (E Y Li BS, Prof C H King MD); 2 Department of Mathematics, Applied Mathematics, and Statistics (Prof D Gurarie PhD, X Zhu BS); 3 Case Western Reserve University, Cleveland, OH, USA; and Department of Medicine, University of California, San Francisco, San Francisco, CA, USA (N C Lo MD)

## Abstract

**Background:**

Schistosomiasis is endemic in many low-income and middle-income countries. To reduce infection-associated morbidity, WHO has published guidelines for control of schistosomiasis based on targeted mass drug administration (MDA) and, in 2017, on supplemental snail control. We compared the current WHO guideline-based strategies from 2012 to an alternative, adaptive decision making framework for control in heterogeneous environments, to estimate their predicted relative effectiveness and time to achievement of defined public health goals.

**Methods:**

In this model-based comparison study, we adapted an established transmission model for Schistosoma infection that couples local human and snail populations and includes aspects of snail ecology and parasite biology. We calibrated the model using data from high-risk, moderate-risk, and lower-risk rural villages in Kenya, and then simulated control via MDA. We compared 2012 WHO guidelines with a modified adaptive strategy that tested a lower-prevalence threshold for MDA and shorter intervals between implementation, evaluation, and modification. We also explored the addition of snail control to this modified strategy. The primary outcomes were the proportion of simulations that achieved the WHO targets in children aged 5–14 years of less than 5% (2020 morbidity control goal) and less than 1% (2025 elimination as a public health problem goal) heavy infection and the mean duration of treatment required to achieve these goals.

**Findings:**

In high-risk communities (80% baseline prevalence), current WHO strategies for MDA were not predicted to achieve morbidity control (<5% prevalence of heavy infections) in 80% of simulations over a 10-year period, whereas the modified adaptive strategy was predicted to achieve this goal in over 50% of simulations within 5 years. In low-risk and moderate-risk communities, current WHO guidelines from 2012 were predicted to achieve morbidity control in most simulations (96% in low-risk and 41% for moderate-risk), although the proposed adaptive strategy reached this goal in a shorter period (mean reduction of 5 years). The model predicted that the addition of snail control to the proposed adaptive strategy would achieve morbidity control in all high-risk communities, and 54% of communities could reach the goal for elimination as a public health problem (<1% heavy infection) within 7 years.

**Interpretation:**

The modified adaptive decision making framework is predicted to be more effective than the current WHO guidelines in reaching 2025 public health goals, especially for high-prevalence regions. Modifications in current guidelines could reduce the time and resources needed for countries who are currently working on achieving public health goals against schistosomiasis.

**Funding:**

University of Georgia Research Foundation, The Bill & Melinda Gates Foundation, and the Medical Scientist Training Program at Stanford University School of Medicine.

## Introduction

Schistosomiasis, a chronic disease caused by parasitic flukes of the genus *Schistosoma*, remains highly prevalent in many low-income and middle-income countries.^1^ The estimated global prevalence of active infections is more than 190 million.^2^ *Schistosoma* reproduces through two different hosts during its lifecycle—an intermediate snail host and a definitive human host.^3^ Three main species cause disease in humans: *Schistosoma mansoni* and *Schistosoma japonicum* cause intestinal and hepatosplenic disease, and *Schistosoma haematobium* causes urogenital pathology.^1^ Schistosomiasis can result in long-term, severe complications that include hepatic, intestinal, ureteric, and bladder fibrosis, as well as bladder cancer. Schistosomiasis is most often treated with praziquantel, which targets adult worms but does not protect the patient against reinfection.^4^ Many schistosomiasis control programmes have reduced local disease prevalence in affected populations with the use of targeted mass drug administration (MDA) delivered as repeated school-based or community-wide treatments. However, prevalence reduction has not been achieved in all treated communities,^5,6^ and, in addition, at-risk areas often have a rebound of infection and disease prevalence after drug treatment efforts are stopped.^7,8^ More effective disease control might ultimately be achieved through environmental modifications that separate humans from contaminated water sources,^9^ or through snail population reductions with molluscicides, as these immediately reduce local snail populations and thus snail-to-human transmission.^10,11^

**Research in context**

**Evidence before this study**

We did not do a literature search before commencement of this comparison study. Periodic mass drug administration (MDA) is the recommended cornerstone policy for control of many helminth infections in endemic regions of the world. Given the large variation in community responses to this type of intervention observed in large-scale randomised trials, there is ongoing controversy regarding whether the WHO goal of morbidity elimination by 2020 is actually feasible for schistosomiasis. We extended our previous work with the Neglected Tropical Disease Modelling Consortium to explore the impact of 2020 treatment guidelines using databases based on detailed populations from coastal Kenya. Analysis by the Consortium has suggested that current WHO guidelines will only achieve 2020 morbidity goals in low-prevalence settings. However, to achieve programme targets in moderate to high-prevalence settings, it is projected that MDA treatment coverage will have to expand to at least 85% for children aged 5–14 years along with treatment of at least 40% of individuals aged 15 years and older. Although current WHO guidance also suggests increasing the frequency of MDA in refractory communities, this switch is recommended only after 5–6 years of unsuccessful results.

**Added value of this study**

This study used improved modelling methods to simulate the effect of both the standard WHO treatment policy and a proposed modified adaptive strategy for MDA implementation in communities with high risk, moderate risk, and low risk of persisting Schistosoma infection. We obtained fairly pessimistic projections regarding outcomes when following current WHO-recommended strategies—increased coverage alone proved unable to achieve WHO morbidity reduction and elimination goals in moderate to high-prevalence settings.

We found that using the standard MDA treatment strategy for more than 2–3 years will lead to apparent stagnation in programme impact, resulting in no further progress towards control. By contrast, for our proposed adaptive strategy, we found that decreasing the time interval between initiation and evaluation, with earlier switching of treatment strategy and lowering of the prevalence threshold for moving to a more aggressive strategy, would lead to more efficient net prevalence reduction. Further addition of snail control projected achievement of morbidity reduction goals (<5% prevalence of heavy infections) in 100% of settings, and achievement of elimination goals (<1% prevalence of heavy infections) in 54% of communities if a three-stage strategy is used.

**Implications of all the available evidence**

Results of this and other forecasting studies strongly suggest the need to alter current WHO treatment guidelines for schistosomiasis control to realistically approach the morbidity and elimination targets set for all communities by 2020 and 2025. Although MDA has been the mainstay approach for schistosomiasis disease control, further implementation research should be done to establish the long-term costs and combined benefits of complementary interventions such as molluscicide use, environmental modification, and behavioural interventions to more effectively reduce disease prevalence worldwide.

**Figure 1:**
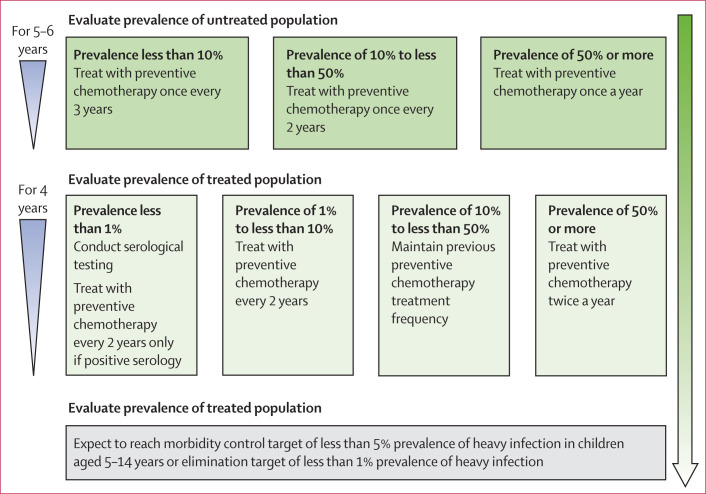
Standard WHO strategy for drug-treatment-based schistosomiasis control Treatment is targeted to the entire population of children aged 5–14 years, but to limit adverse reactions, children who are acutely ill are excluded. Coverage levels are estimated after each cycle of treatment.^12^

In the past 20 years, WHO has fostered resources, donors, and partners to help in the control and elimination of many neglected tropical diseases, including schistosomiasis. Current WHO guidelines from 2012 (figure 1) include a set of guidelines for morbidity control, defined as reaching a prevalence of less than 5% heavy intensity *Schistosoma* infections among local children aged 5–14 years, referred to as school age children in WHO guidelines.^13^ WHO’s strategy for schistosomiasis control suggests treatment on the basis of disease prevalence among children aged 5–14 years in a given population, aiming for a minimal treatment coverage of 75% of children aged 5–14 years.^12^ Heavy intensity infection is defined as 50 eggs or more per 10 mL urine for *S haematobium* infection, and 400 eggs or more per gram faeces for *S mansoni* and *S japonicum* infections. In addition, elimination of schistosomiasis as a public health problem has been operationally defined as having less than 1% prevalence of heavy infections in children aged 5–14 years. The target for reaching morbidity control has been set for 2020^12^ and that of elimination as a public health problem has been set for 2025. The associated guidelines rely heavily on targeted MDA as the main control strategy.^14^ Most schistosomiasis control programmes are focused on morbidity reduction and are mainly treating subpopulations of children aged 5–14 years via school-based or occasionally, community-based distribution. However, in 2017, large-scale operational research trials identified limitations to both forms of drug-treatment-only control strategies.^5,6^ The emergence of so-called persistent hot spots in the face of continued MDA implementation (as recommended) in endemic regions calls for reassessment and possible revision to global implementation guidelines.^15^

Previous modelling done by the Neglected Tropical Disease Modelling Consortium^16^ found that the WHO goals for morbidity control and elimination as a public health problem are likely to be attainable in low-prevalence but not moderate to high-prevalence settings using current guidelines. The modelling consortium furthermore recommended increasing treatment frequency or coverage to 85% of children aged 5–14 years and 40% of individuals aged 15 years and older to reach these goals for moderate to high-prevalence settings. In the current study, using the stratified worm burden modelling method, we examined whether the current guidelines could achieve their key public health targets in low-transmission, moderate-transmission, and high-transmission communities, and if so, how long it would take to reach these targets. We then compared the performance of the current guidelines to a newly proposed adaptive strategy based on the Modelling Consortium’s recommendations to establish how to optimise the duration of each treatment stage and the time interval between shifts in treatment intensity, and the likelihood of successfully achieving targeted control levels given known variability in inputs. We then explored the role of local environmental and behavioural exposures and their effect on disease control in terms of fostering persistent hotspots of transmission.

## Methods

### Model overview

In this model-based comparison study, we adapted an existing transmission model for schistosomiasis—the stratified worm burden model.^17–20^ This model produces simulated egg count data for hypothetical low-transmission, medium-transmission, or high-transmission villages for the model set-up. During calibration, initial model outputs are compared with observed egg count data from field studies to generate posterior distribution of probable parameter values for human in-host worm dynamics and human–snail transmission parameters. Environmental uncertainty is also incorporated when generating the calibrated model. For control strategy simulation, multiple different control strategies are applied to the calibrated models to predict outcomes of various treatment methods for each community population.

The model was fitted to a specific dataset from coastal Kenya,^21–23^ which contains a range of endemic community settings (including high-level, moderate-level, and low-level transmission). The model simulated the parasite dynamics in both the human and snail populations in individual communities with the target goals of morbidity control (<5% prevalence of heavy infections among children aged 5–14 years) and elimination as a public health problem (<1% prevalence of heavy infections among children aged 5–14 years) defined at the single community level. It also took into consideration intra-host worm biology (ie, mating, aggregation, and random egg release), snail population biology,^23^ and other environmental uncertainties such as transmission parameters and relative contact rates for different age groups.^24^ We chose a deterministic structure given that the programme focused on public health control, and not elimination.

We simulated control implementation according to both the current WHO guidelines and the proposed adaptive guidelines to compare the length of time required and proportion of simulations that could achieve the 2020 WHO morbidity control goal (<5% prevalence of heavy infections among children aged 5–14 years) and its 2025 elimination as a public health problem goal (<1% prevalence of heavy infections among children aged 5–14 years). For the current WHO guidelines from 2012, we modelled both the suggested coverage value of 75% in children aged 5–14 years and the enhanced coverage values (parameter values for the model are displayed in the appendix p 5).

### Model structure

#### Human factors

**Figure 2:**
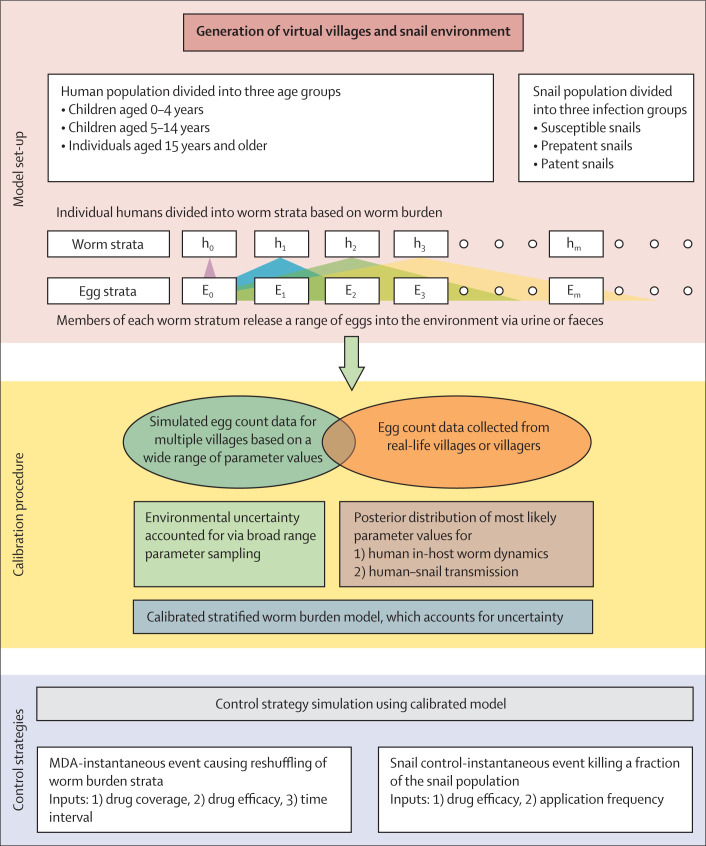
Stratified worm burden modelling approach MDA=mass drug administration. h=human. E=egg. m=placeholder signifying integral sequence.

The modelled human population was divided into three age groups: children aged 0–4 years, children aged 5–14 years, and individuals aged 15 years and older (figure 2). In the stratified worm burden system, worms were acquired or lost at certain age-specific rates, and each host group was subdivided into worm-burden strata.^17–20^ Following age-specific calibration of transmission parameter values, each group was characterised by its age-specific worm burden distribution.

MDA was simulated as an instantaneous event that leads to the elimination of a certain fraction of the worms within each human worm-burden stratum, resulting in a reshuffling of strata in each treated age bracket—generally moving individuals from higher to lower burden strata. This redistribution was then translated into a predicted prevalence and mean age-intensity. Key inputs for MDA were the targeted population subgroup, their coverage level, estimated drug efficacy, and the frequency of treatments. Similarly, we also assumed that molluscicide application removed a proportion of the snail population, which was incorporated into the efficacy of the application event in terms of reducing the process of reinfection.

#### Snail environmental variables

Previous work has shown that dynamic modelling of transmitting snail populations is essential for accurate prediction of transmission in the face of environmental uncertainties.^25^ In the present study, we assumed that the snail population manifested logistic growth with a high maximal reproduction rate but a prescribed environmental carrying capacity (K). We divided the snail population into three groups on the basis of their infection status—susceptible (snails that had not been infected), prepatent (infected but not yet releasing *Schistosoma* cercariae), and patent (infected and releasing cercariae). Because of the absence of extensive data for these factors, we used a broad range of snail environmental inputs in the simulations to account for this uncertainty.

We include a non-linear (saturated) relationship between human egg output and the force of snail infection in the models, on the basis of previous work showing the non-linear approach has resulted in better model prediction.^18,19,26^ Notably, non-linear force of infection is associated with a stronger post-MDA infection rebound than the traditional linear force of infection models. This difference substantially affects the long-term projections of control programme outcomes in higher-risk settings.^26^

#### Public health strategies

**Figure 3:**
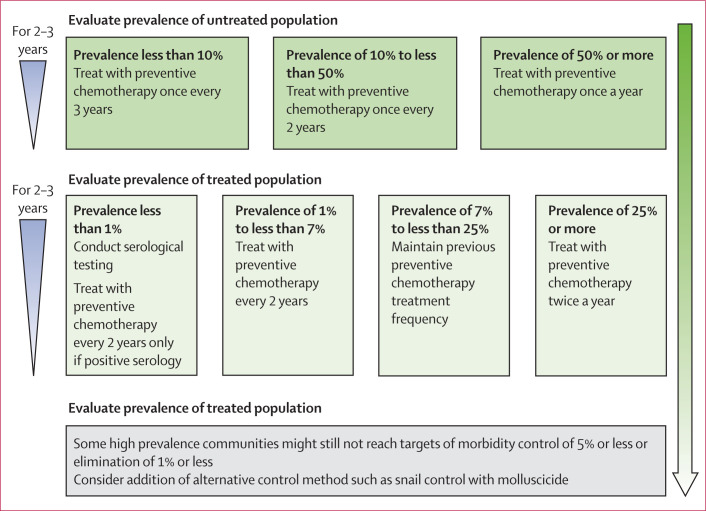
Modified adaptive strategy for schistosomiasis morbidity control The added modifications are: (1) to decrease the time interval between initiation and evaluation of treatment strategy outcomes to 2–3 years due to projected stagnation of further treatment impact, and (2) to lower the prevalence threshold for switching from annual to biannual treatment strategies. Although we chose to use 7% and 25% as the cutoff thresholds during the changeover year, these values are not rigid and could be adjusted as needed for local conditions.

In the analysis of treatment outcomes for different intervention protocols, we compared the current WHO strategy from 2012 (figure 1) with a proposed modified adaptive strategy (figure 3). The current WHO guidelines suggest choosing the initial treatment frequency on the basis of baseline prevalence and then re-evaluating after 5–6 years of drug delivery. Depending on the prevalence after this re-evaluation, the community then receives a possible change in frequency of chemotherapy delivery. This is re-evaluated again after 4 more years of treatment to see if programme targets have been reached. The proposed modified adaptive strategy affects assessment earlier, after two or three rounds of MDA, to more quickly identify communities with high persistent force of infection, allowing for earlier implementation of more aggressive treatment coverage or frequency of administration, or both. Subsequent early assessment of the effect of enhanced MDA then also permits identification of locations where supplemental snail control can be of most benefit.

#### Uncertainty and sensitivity analysis

Uncertainties in both aspects of human and snail biology and exposure factors are taken into account via the stratified worm burden model. For the human component of the coupled system, posterior distributions of probable parameter choices were generated through comparison of the simulated test data to eggs per 10 mL urine datasets for *S haematobium* collected from communities in coastal Kenya. This method allowed for the generation of ensembles of parameter choices that were weighted to more accurately reflect real-world human processes. Further seasonal and environmental snail uncertainty was taken into account through the random sampling of the so-called snail parameter space, as environmental data are scarce and environmental inputs tend to be specific to location and situation. This approach simulated a wide range of geographical and environmental inputs. Thus, the predicted outcomes can be viewed as an ensemble of near-identical host communities (in terms of baseline human infection) placed in a wide range of diverse snail environments (see appendix pp 5–7 for further discussion on sensitivity and uncertainty).

### Data inputs

Model communities were calibrated using anonymised population-wide *S haematobium* infection datasets collected from 12 villages in the Msambweni region of coastal Kenya.^21–23^ In those surveys, village residents were tested for the parasite via duplicate filtration of 10 mL midday urine and egg counting by light microscopy. For the analysis, we divided these villages into three categories according to infection prevalence levels in children aged 5–14 years as per WHO guidelines:^13^ high (prevalence >50% in children aged 5–14 years), moderate (prevalence >10% but <50% in children aged 5–14 years), and low (prevalence <10% in children aged 5–14 years) prevalence villages.

### Role of the funding source

The funder of the study had no role in study design, data collection, data analysis, data interpretation, or writing of the report. The corresponding author had full access to all the data in the study and had final responsibility for the decision to submit for publication.

## Results

**Figure 4:**
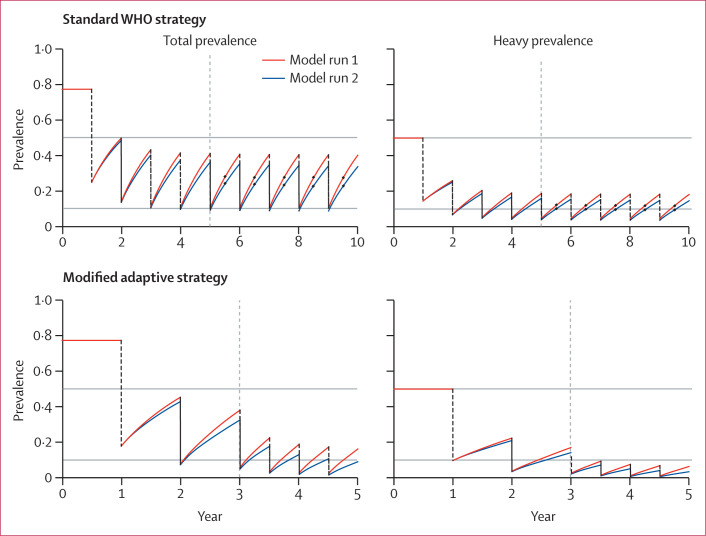
Comparison of Schistosoma haematobium prevalence histories under standard WHO control strategy to those under modified adaptive strategy for high-prevalence communities Total prevalence levels and heavy infection prevalence (>50 eggs per 10 mL urine) levels in children aged 5–14 years are shown. The grey vertical dotted line shows strategy re-evaluation and possible switch depending on guideline recommendations, which occurred at year 5 for the standard WHO control strategy and year 3 for the modified adaptive strategy. Red and blue lines show two separate iterations of the model with the same initial values. Discrepancy between the two iterations shows the effect of modeling uncertainty (eg, environmental variation) for the same community. The grey horizontal lines indicate 50% and 10% prevalence.

Simulated MDA response patterns for both strategies indicated sharp post-treatment drops in infection prevalence followed by ongoing transmission-related rebound (figure 4). The strength of the rebound was dependent on the transmission intensity and local snail ecology.^26^ For any calibrated system, the strength of infection rebound relative to drug-mediated reductions in prevalence determined the progress of the MDA regimen over time. We found a rapid reduction of infection after only two to three MDA rounds, followed by transition into a stagnated (limit cycle) pattern of reinfection that prevents further progress. At that point, the prevalence decrease obtained from each cycle of MDA is balanced by a rebound in infection prevalence, leading to no further gain (figure 4; see appendix p 8 for details on stagnation)

**Figure 5:**
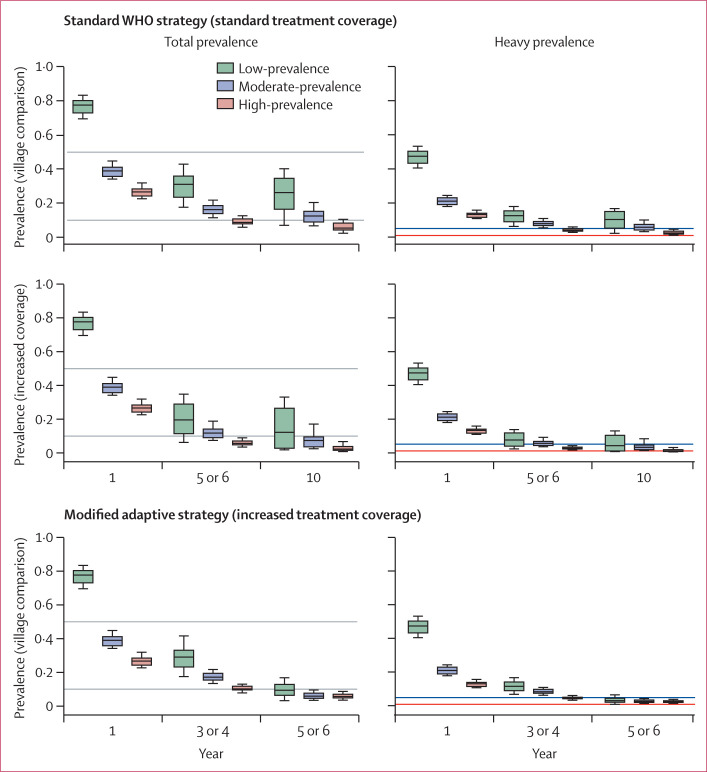
Effectiveness in achieving morbidity control and elimination goals for Schistosoma haematobium under standard WHO control strategy and modified adaptive strategy for high, moderate, and low-prevalence communities Total prevalence and heavy infection prevalence (>50 eggs per 10 mL urine) in children aged 5–14 years are shown. Standard treatment coverage is 75% among children aged 5–14 years; increased treatment coverage is 85% in children aged 5–14 years and 40% in those aged 15 years and older. 2020 goals of morbidity reduction (<5% prevalence of heavy infection among children aged 5–14 years) are displayed by the blue horizontal lines and elimination as a public health problem (<1% prevalence of heavy infection among children aged 5–14 years) goals are displayed by the red horizontal lines, in the right-hand panels. Box-whisker charts show the range of possible outcomes (due to input uncertainty) for low, moderate, and high-prevalence villages. Re-evaluation for the standard guidelines is shown at year 5 for high-prevalence villages and year 6 in low and intermediate-prevalence villages. For modified WHO guidelines, re-evaluation occurs at year 3 for high-prevalence and year 4 for low and moderate-prevalence villages. Quartiles are different between WHO standard and modified strategies for year 1 due to modelled uncertainty. The grey horizontal lines indicate 50% and 10% prevalence.

In high-prevalence communities, the study simulations consistently found that current WHO guidelines from 2012 predicted early stagnation and difficulty in reaching stated public health goals within the desired 5–10-year time frame (figure 5). This finding was robust even for increased MDA coverage values of 85% for children aged 5–14 years and 40% for individuals aged 15 years and older. In low-prevalence villages (prevalence of <10% for children aged 5–14 years), the model predicated a greater likelihood of reaching morbidity control and elimination as a public health problem targets than in higher-prevalence villages. However, there was a failure to eliminate as a public health problem in the same programmatic time frame, regardless of the initial prevalence of the village (figure 5). The model predicted that the modified adaptive strategy was able to reach morbidity control (<5% prevalence of heavy infections in children aged 5–14 years) for more than half of the simulations of the high-prevalence villages in around 5 years (figure 5).

Although the standard WHO strategy was predicted to be partly successful in communities with a moderate starting infection prevalence, only 41% of these communities would reach the morbidity control target with a treatment coverage of 75% among children aged 5–14 years, and 78% of communities would reach the morbidity control target with increased treatment coverage (figure 5). We found that similar results could be reached in a shorter amount of time if earlier schedule switching was used (eg, changing to MDA every year instead of every 2 years). It was predicted to be possible to reach control targets using standard WHO protocols in a low-prevalence community. However, the modified adaptive strategy showed the possibility of reaching these targets in a shorter time frame.

**Figure 6:**
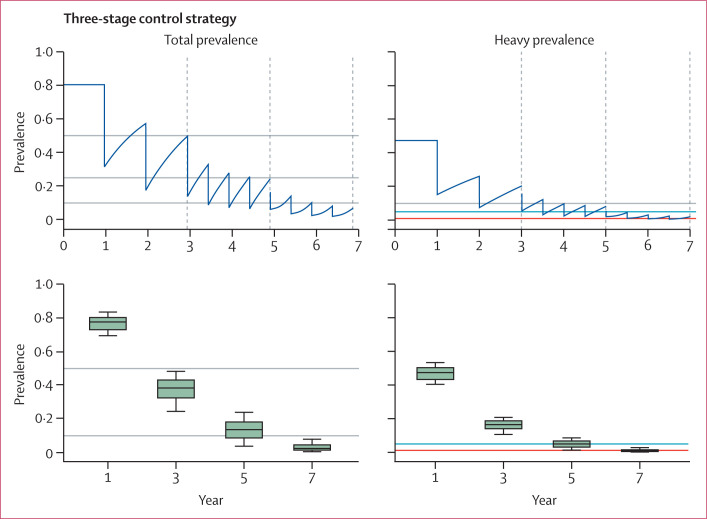
Three-stage control strategy with addition of snail control to MDA in the third step Effects of a three-stage control strategy are shown in which the modified adaptive strategy is used for the first two steps. Snail control is then added 2 years after the second year of intensified MDA. Note the addition of snail control leads to a slower rebound of infection prevalence. The upper graphs show the history of prevalence values for children aged 5–14 years given treatment implementation strategy, and the lower box-whisker charts display the range of possible results. The WHO goal of morbidity reduction (<5% prevalence of heavy infection among children aged 5–14 years) is displayed by the blue horizontal lines and elimination as a public health problem (<1% prevalence of heavy infection among children aged 5–14 years) goals are displayed by the red horizontal lines, in the right-hand panels. The grey vertical dotted lines show the points where there was re-evaluation and strategy switching in the modified adaptive strategy. The grey horizontal lines indicate 50% and 10% prevalence. MDA=mass drug administration.

Finally, we modelled the addition of a snail control intervention—ie, focal treatment of human water contact sites with molluscicide to suppress transmitting snail populations. We incorporated a three-stage control strategy, in which the modified MDA scheduling could be augmented, if needed, with snail control. In this enhanced intervention strategy for high-risk communities, the initially implemented annual MDA was followed by a year 3 switch to a more intense twice-yearly regimen as shown in figure 2. After 2 years of biannual drug treatment, snail control was then added (figure 6). This change predicted a further decline in prevalence and avoidance of programme impact stagnation. Even when modelling uncertainty was taken into account, 100% of simulated villages were predicted to reach a heavy infection prevalence level of less than 5%, and 54% of the villages were able to achieve WHO elimination as a public health problem goals of less than 1% heavy infections by year 7 (figure 5).

## Discussion

In this simulation-based study, we predicted that achieving schistosomiasis morbidity reduction targets (<5% prevalence in children aged 5–14 years) with current WHO guidelines from 2012 was unlikely in high-prevalence settings, and that a modified adaptive strategy could be more effective in achieving public health targets. In low-risk and moderate-risk communities, the adaptive strategy achieved results similar to the current WHO strategy, but in a shorter time interval, with fewer cycles of MDA. Within the simulations, we observed that a repeated implementation of standard WHO-guidelines-based MDA led to an initial reduction in prevalence within two to three cycles. However, further MDA cycles did not continue the decline in infection prevalence, resulting in a failure to achieve elimination as a public health problem. With the suggested modified adaptive guidelines, we found that programmes could achieve a larger decrease in prevalence over a shorter time period, employing fewer rounds of MDA. For high-risk communities, this modified implementation strategy predicted a greater likelihood of success than protocols based on the current WHO guidelines from 2012.

These predictions assume ideal conditions regarding MDA and snail control strategies, although they do incorporate many heterogeneities between settings. Although we assumed a minimal coverage level of 75–85% in children aged 5–14 years and 40% for individuals aged 15 years and older, the true coverage of each age group might widely fluctuate in the real world due to variations in drug access and delivery and the surrounding social and political climate. Drug delivery and education are usually done through a coordinated effort between programme managers, other staff, and local members of the community. Each location might have a different method of handling drug delivery, which could affect the timing and likelihood of a community receiving the drug. In addition, the percentage of children treated often fails to reach the anticipated coverage value. In the first couple of years of MDA treatment in Malawi, less than half of children took the medication because they were suspicious of it. In addition, some community members considered the disease to be normal and would keep their children home during treatment days.^12^ This finding highlights the importance of community education programmes to enhance participation to levels that can limit the local force of reinfection. In the simulations that were previously published, potentially optimistic values were assumed for efficacy of both drug treatment and molluscicide.^27^ Treatment failure after an MDA or snail control cycle might be more probable than we have predicted, meaning that even fewer villages will reach morbidity control and elimination as public health problem goals. The precision of the predictions should be taken with caution, but we believe that they can be used to inform future policy changes.

Although millions of people have been treated via WHO control programmes,^28^ global schistosomiasis prevalence is still high,^2^ as the parasite has many factors that favour its transmission. The participation of both human and snail hosts allows for two stages of parasite increase. Because of asexual reproduction in the snail, a small percentage of affected snails can result in the release of many infectious cercariae, which, in turn, can infect multiple exposed humans. In this regard, *Schistosoma* parasites are very different from insect-borne helminths such as filaria, *Onchocerca*, and *Loa loa*, and soil-transmitted helminths such as *Ascaris* and hookworm. Policy makers often assume that the approach to elimination of all of these vector-borne and soil-transmitted diseases lies along the same implementation pathways (ie, MDA) and general approach of implementation. Past programmatic experience has shown that cessation of a schistosomiasis treatment programme often leads to a rapid return to previous disease prevalence levels.^7,8^ Therefore, it is crucial to design a sustainable control programme that can maintain government support, adequate funding, community involvement, coordination between control programmes, with frequent critical assessment of progress.^29^ Increased availability of praziquantel has allowed greater flexibility in MDA treatment strategies. An emerging concern for *Schistosoma* transmission control is the issue of persistent hotspots—ie, villages that do not respond well to MDA in terms of reductions in prevalence and intensity of infection.^5,6^ The question as to why some villages respond when others do not still needs to be explored, and strategies for early detection are urgently needed. For all of these reasons, new guidelines that consider adaptive strategies including an earlier change in strategy for updating the optimal MDA or public health strategy for schistosomiasis could yield great public health impact. However, costs related to drug distribution and prevalence monitoring must be considered in programme design. Our earlier work^17^ has shown the potential benefits of integrated environmental snail control versus MDA alone in terms of cost-effectiveness, and we found that combined MDA and snail control becomes the strategy of choice in high-transmission areas. Further analysis regarding the cost-effectiveness of the adaptive strategy applied to a mixed-risk multi-village setting still needs to be done, which goes beyond earlier cost-effectiveness work that optimised the prevalence threshold for MDA strategies.^30^

Our study model takes into account a broad range of uncertainties about the potential variability of human test data and the differences in snail environment. Because the data regarding snail and environmental factors are often missing from programme monitoring and evaluation, we simulated a wide range (in some sense, the full range) of potential environmental inputs. These represented an ensemble of virtual communities placed in different environments and subjected to the same strategies. We then estimated the probability of reaching WHO targets for control.^13^ Because the inputs are not tied to any particular region, environment, or village structure, they provide a useful perspective on implementation across a range of communities with similar baseline levels, varying from high transmission to low transmission, in different environments with different *Schistosoma* species.

The results of this study should be interpreted within the limitations in model assumptions and study data. There are substantial aspects of parameter and structural uncertainty in the model, which we addressed in part through random sampling of relevant parameter ranges. The most important include estimates for drug efficacy, which was inferred from worm clearing rates and environmental uncertainties. The environmental uncertainties include infected and patent snail densities, relative snail to human abundance, and the probability of snail infection per unit of host contagion. In the absence of such data, we used broad ranges of environmental inputs consistent with observed MDA responses in field studies. Our reported estimates for target reduction thus account for combined uncertainty of model calibration (on the basis of human test data), and a broad range of environmental uncertainties. In the future, more accurate human diagnostics and snail data could potentially narrow this range of uncertainty. We also assumed homogeneous mixing within each subpopulation for transmission, although this is a simplification of the real-world process.

Although the modified adaptive strategy outperformed current WHO guidelines from 2012, the changes will require some thought for implementation and further model development. The analysis was done in the context of isolated coupled human–snail communities, although connectivity (human and snail movement between communities) is known to play an important role in dynamics of transmission and control in some areas.^31–33^ Furthermore, we assumed some parameters to be constant in our model despite there being realistic epidemiological and environmental changes. We assumed a stationary transmission environment in the analysis; however, in many places the transmission environment can be highly seasonal with large variations in snail populations and human–snail contacts.^25^ There might also be individual variation regarding the efficacy of praziquantel, which was not shown in the model. Likewise, treatment coverage and systematic compliance levels could vary across time and location due to economic, political, cultural, and logistical factors. Further field validation of these findings and consideration of cost-effectiveness should be undertaken as part of public health implementation.

Regarding the aspirational goal of elimination as a public health problem, for high-risk communities, even modified implementation strategies with MDA alone did not achieve elimination in the majority of the simulated villages. No matter how much progress was achieved by the end of the programme, the model predicted a gradual rebound of infection to pre-control levels (see appendix p 11 for further details on rebound).^8,23^ Although the intensive adaptive regimen augmented with snail control for an additional 2–3-year period might attain the desired elimination as a public health problem goal, any progress can be complicated by the risk of reintroduction of infection from outside sources, whether through humans or snails. It will be necessary, therefore, to take a further look at so-called post-elimination strategies (ie, monitoring, evaluation, and control strategies) that would allow the community to sustain very low-prevalence levels before complete interruption of *Schistosoma* transmission. In summary, a modified adaptive strategy for MDA, with or without snail control, will probably be more effective than the current WHO guidelines from 2012 in achieving key public health goals, especially in high-risk communities that emerge as persistent hotspots of infection.

### Contributors

EYL, DG, NCL, XZ, and CHK designed the research. EYL, DG, XZ, and CHK did the research. EYL, XZ, DG, and NCL contributed new analytic tools. EYL, DG, NCL, and CHK analysed data. EYL, DG, NCL, XZ, and CHK wrote the paper.

### Declaration of interests

DG and CHK report grants from Schistosomiasis Consortium for Operational Research and Evaluation, during the conduct of the study. NCL reports personal fees from WHO, outside the submitted work.All other authors declare no competing interests.

### Data sharing

Data used for this analysis are available from CHK (chk@case.edu).
